# Predictive value of dynamic change of haemoglobin levels during therapy on treatment outcomes in patients with Enneking stage IIB extremity osteosarcoma

**DOI:** 10.1186/s12885-018-4279-8

**Published:** 2018-04-16

**Authors:** Jian Tu, Lili Wen, Zijun Huo, Bo Wang, Yongqian Wang, Hongyi Liao, Weihai Liu, Xian Zhong, Jianqiu Kong, Mengqi Wang, Gang Huang, Junqiang Yin, Xianbiao Xie, Jingnan Shen

**Affiliations:** 1 Department of Musculoskeletal Oncology, First Affiliated Hospital of Sun Yat-sen University, Guangzhou, 510080 China; 2 Department of Anesthesiology, Sun Yat-sen University Cancer Center, Guangzhou, China; 3 Department of Endocrinology, First Affiliated Hospital of Sun Yat-sen University, Guangzhou, China; 4 The eight year program, Zhongshan School of Medicine, Sun Yat-sen University, Guangzhou, China

**Keywords:** Osteosarcoma, Haemoglobin, Dynamic change, Prognostic factor

## Abstract

**Background:**

We aimed to investigate the roles of hemoglobin (Hb) concentrations and dynamic change during treatment on outcomes of patients with extremity osteosarcoma.

**Methods:**

We retrospectively analysed 133 patients with Enneking stage IIB extremity osteosarcoma who underwent standard treatments, including univariate and multivariate analyses of patient charateritics, Hb concentrations and changes during pretreatment, neoadjuvant, adjuvant chemotherapy, and decreased Hb levels (ΔHb) to assess their prognostic value in 5-year overall survival (OS) and lung metastasis-free survival (LMFS).

**Results:**

Five-year OS or LMFS were similar between patients who were anaemic and non-anaemic during pretreatment, neoadjuvant or adjuvant chemotherapy. Patients with continuously decreasing Hb had lower 5-year OS (52.3%) than those without continuous Hb decrease (68.5%, *P* = 0.04). Patients with ΔHb > 7.6 g/L had lower 5-year OS (57.5%) than those with ΔHb ≤7.6 g/L (75.8%, P = 0.04). However, continuous Hb decrease had no prognostic effect on 5-year LMFS. Subgroup analyses showed that patients who were anaemic during pretreatment, neoadjuvant, or adjuvant chemotherapy with ΔHb ≤7.6 g/L had better outcomes than those with ΔHb > 7.6 g/L (*P* < 0.05, for both).

**Conclusion:**

Dynamic Hb decrease and ΔHb > 7.6 predicted poor5-year OS in patients with Enneking stage IIB extremity osteosarcoma. Attempts to correct anaemia and their effects on outcomes for osteosarcoma patients should be investigated in future trials.

## Background

Osteosarcoma is the most common solid bone malignancy in children and adolescents. With the integration of neoadjuvant, adjuvant chemotherapy and surgery, the 5-year overall survival (OS) rate has increased to 50–70% for non-metastatic extremity osteosarcoma. However, an improvement of OS has been sparse in the last 2 decades, although clinicians have tried to combine different chemotherapy agents to achieve better survival outcomes [1]. Many hypotheses account for the lack of improvement in OS. However, the lack of individual risk stratification and tailored therapy for osteosarcoma patients may partly account for this disappointing progress.

Haemoglobin (Hb) levels are reported to be a prognostic factor in many malignancies [2–5] with low pretreatment Hb level shown to predict poor outcomes in most studies. Lower Hb concentration may cause a decline of blood oxygen supply and tumour hypoxia, which may contribute to the development of treatment resistance [6, 7]. Additionally, dynamic Hb changes during treatment are reportedly better survival predictors for nasopharyngeal carcinoma [8, 9]. Osteosarcoma patients are widely thought to benefit from high doses chemotherapy of long duration. As chemotherapy resistance is a major cause of treatment failure [10]. Changes in Hb during neoadjuvant and adjuvant chemotherapy for osteosarcoma patients may be a fruitful area of study; few data regarding the prognostic value of Hb levels for osteosarcoma patients have been available.

In this study, we investigated whether Hb levels and differences in decreased Hb levels during therapy could predict the survival outcomes of patients with Enneking stage IIB extremity osteosarcoma.

## Methods

### Patients and treatment

Patients with Enneking stage IIB extremity osteosaroma who were treated in the First Affiliated Hospital of Sun Yat-sen University between 2003 and 2010 were enrolled in this retrospective study. Written informed consent for participation in the study was obtained from participants or their parents. This study was approved by the Institutional Ethical Board of the First Affiliated Hospital of Sun Yat-sen University.

**Fig. 1:**
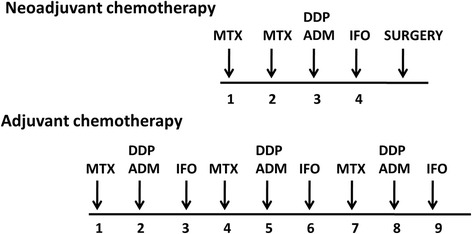
The chemotherapeutic agents and the treatment protocol of 133 patients

Needle biopsies were performed when potential patients presented themselves in the clinic, followed complete blood tests, magnetic resonance imaging of lesion site, computed tomography scan of lung and emission computed tomography of whole body before treatments. The treatment protocol is shown in Fig. 1, and was described in our previous study [11]. The chemotherapy protocol included methotrexate, cisplatin, doxorubicin, and ifosfamide, which are the most commonly used agents in practice.

Blood transfusions were administered if the Hb level was < 70 g/L. No epoetin alfa was used for osteosarcoma patients in our centre.

The inclusion criteria were (a) pathologically confirmed osteosarcoma; (b) tumours that had originated in the extremities without lung metastasis at diagnosis; (c) availability of complete Hb levels records; and (d) standard treatment received as described above. Patients with a Karnofsky Performance State score < 70, older than 40 years or incomplete clinical data were excluded.

### Definition of anaemia and Hb groups

Patients younger than 14 years of age or males were considered anaemic if theie Hb level was < 120 g/L, or < 110 g/L for females older than 14 years, according to the World Health Organization’s definition of anaemia.

Hb baseline level (pretreatment Hb level) was measured before neoadjuvant chemotherapy for all patients. During treatment, Hb level was assessed at the beginning of every chemotherapy cycle, 1 day before surgery, and every 3 days after surgery for 1 week. If the results were abnormal, Hb level would be examined every 2 days. The neoadjuvant Hb level was defined as the average of all Hb concentrations during neoadjuvant chemotherapy. The adjuvant Hb level was defined as the average of all Hb concentrations during adjuvant chemotherapy. Continuous Hb decrease was defined as Hb baseline level > neoadjuvant Hb > adjuvant Hb. The individual difference in Hb value (ΔHb) was equal to the Hb baseline level minus the adjuvant Hb level.

### Follow-up

After completing treatment, all patients were followed up every 3 months for Years 1 and 2, every 4 months for Year 3, every 6 months for Years 4 and 5, and yearly thereafter. X-ray examinations of the lesion site and computed tomography scans of the lungs were performed for every clinic visit in the clinic; emission computed tomography of the whole body would be performed if potential metastasis or relapse was noticed.

### Statistical analysis

SPSS (version 19.0, Chicago IL) statistical software was used for the statistical analysis. OS was defined as the time from diagnosis to death from any cause or the last follow-up visit. Lung metastasis-free survival (LMFS) was defined as the time from diagnosis to detection of lung metastasis for patients with no lung metastasis at the time of initial diagnosis.

The Kaplan-Meier method was used to calculate survival curve; differences between curves were evaluated by the log-rank test. Univariate and multivariate analyses tested the prognostic value of potential factors. Variables found to be significant in the univariate analysis were selected for inclusion in the multivariate analysis with the Cox proportional hazard model. *P* value < 0.05 was considered significant.

The optimal cut-off value for ΔHb was measured by the receiver operating characteristic (ROC) curve. The best threshold was the minimal distance to the ideal point on the ROC curve, for which Se(*c*) + Se(*p*) - 1 was maximized. The total population of this study was then stratified according to this best cut-off point [12].

## Results

### Patient characteristics

**Fig. 2:**
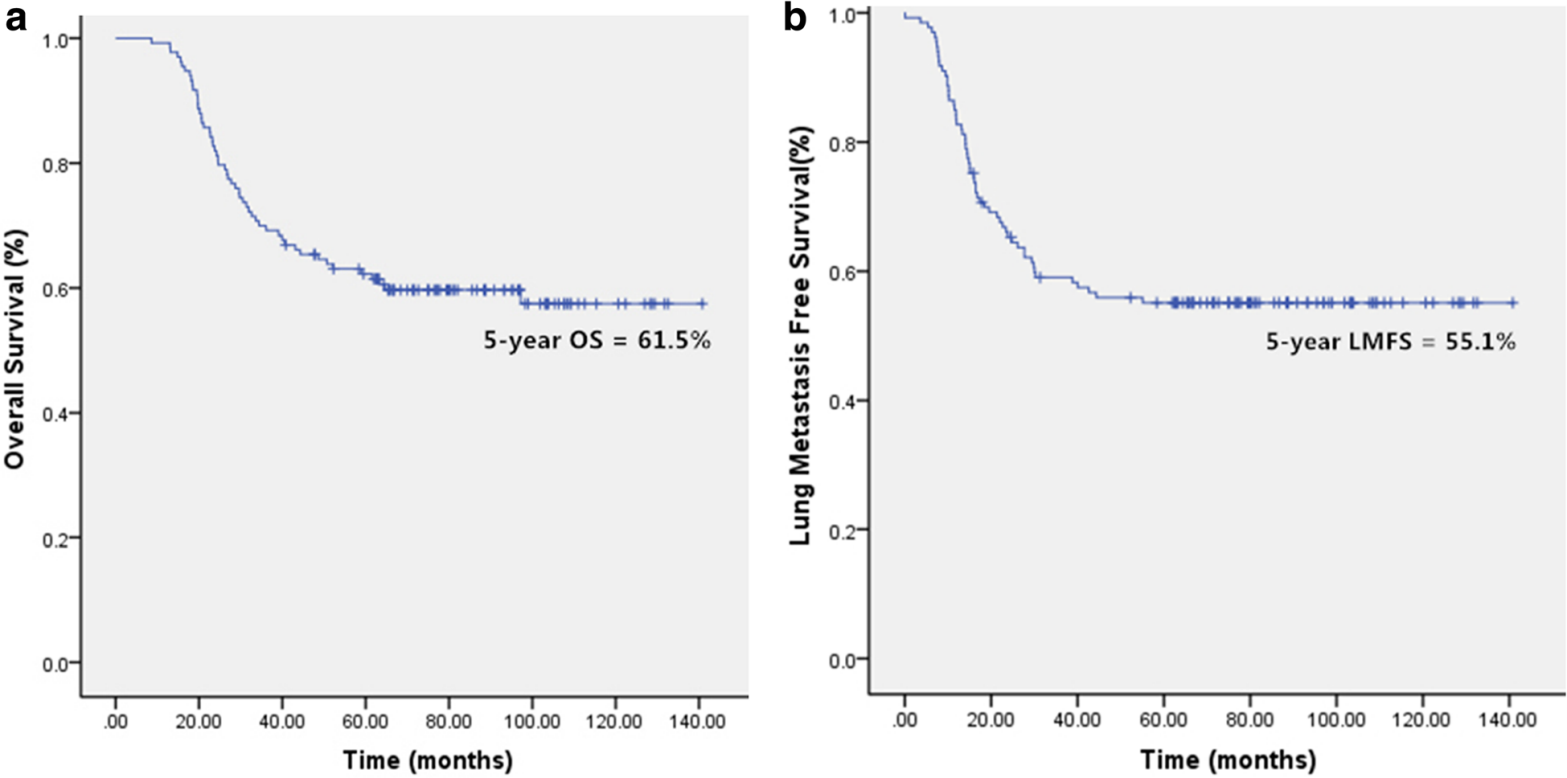
Kaplan-Meier curves showing the overall survival (OS) (**a**) and the lung metastasis free survival (LMFS) (**b**) of the included patients

We retrospectively reviewed and analysed 133 patients with Enneking stage IIB extremity osteosarcoma who had received standard treatment. Their characteristics are shown in Table 1. Their median follow-up time was 62.7 months (range: 8.5–132.3 months). Their 5-year OS was 61.5% (Fig.2a ), and LMFS was 55.1% (Fig. 2b) by Kaplan-Meier survival analysis.

**Table 1:** Characteristics of the included patients

Variables	Patient (n)	%
Age (y)		
< 14	42	31.6
≥ 14	91	68.4
Gender		
Male	91	68.4
Female	42	31.6
Primary site		
Femur	74	55.3
Tibia	37	27.6
Fibula	10	7.2
Humerus	9	6.6
Radius	3	3.3
Enneking stage		
IIB	133	87.5
Pathological fracture		
No	122	92.1
Yes	11	7.9
Histological type		
Osteoblastic	106	79.6
Chondroblastic	19	14.5
Fibroblastic	5	3.9
Others	3	2
Tumor size		
≦8 cm	65	48.9
>8 cm	68	51.1
Surgery type		
Amputation	33	25.0
Limb sparing	100	75.0

The mean pretreatment Hb level was 130.9 g/L (median 131.0 g/L; range: 71.0–162 g/L). Of the 133 patients, 39 (29.2%) were diagnosed as having anaemia before any treatment. Their mean neoadjuvant Hb concentration was 117.2 g/L (median: 117.5 g/L, range: 68.5–149.0 g/L). We found 99 patients (74.4%) to have anaemia during neoadjuvant chemotherapy. Their mean adjuvant Hb level was 114.8 (median 114.5 g/L; range: 70.2–151.2 g/L). During adjuvant chemotherapy, 98 patients (73.7%) were found to be anaemic. According to Hb levels during the therapy period, 68 (44.7%) patients were classified into the Hb continuously-decreased group and 84 (55.3%) patients into the non-continuously decreased group.

### ΔHb cut-off value

The area under the ROC curve for ΔHb was 0.60 (95% CI: 0.51–0.70; *P* = 0.04). The ΔHb cut-off value was 7.6 g/L (85.2% sensitivity; 31.6% specificity). We divided 33 (24.8%) patients into the ΔHb ≤ 7.6 group, and 100 (75.2%) into the ΔHb > 7.6 group.

### Treatment outcomes according to Hb levels

**Fig. 3:**
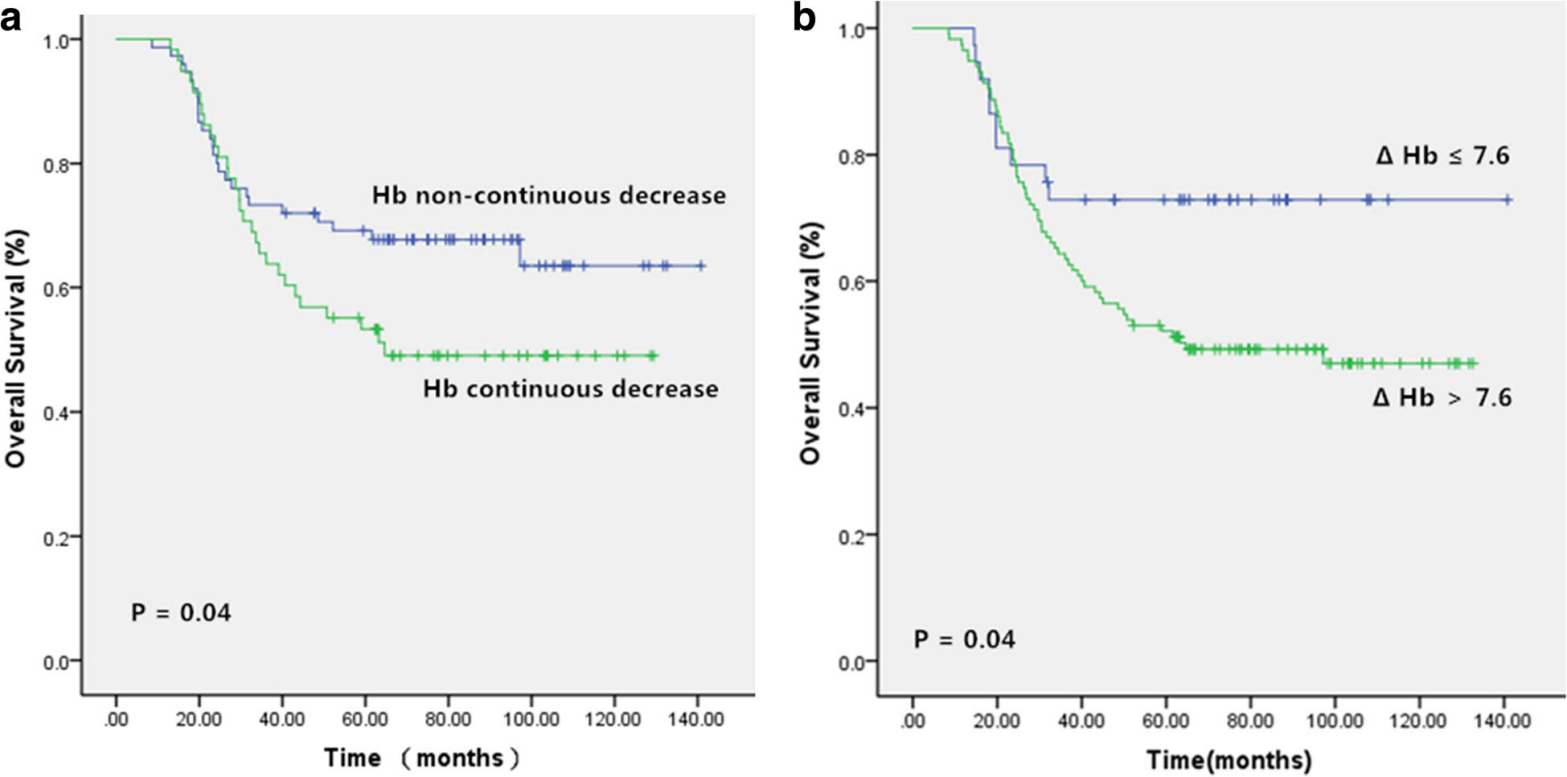
Kaplan-Meier curves showing the overall survival (OS) in Hb continuously decreased and noncontinuously decreased groups (**a**), ΔHb ≤7.6 g/L and ΔHb > 7.6 g/L groups (**b**)

Survival outcomes at different Hb levels are shown in Table 2. The anaemia and non-anaemia groups did not significantly differ in 5-year OS. Patients with continuously decreased Hb had worse 5-year OS than those with non-continuous Hb decrease (52.3% vs. 68.5%, P = 0.04, Fig. 3a). Additionally the ΔHb ≤ 7.6 and ΔHb > 7.6 groups significantly differed in 5-year OS (75.8% vs. 57.5%, P = 0.04, Fig. 3b).

**Table 2:** Treatment outcomes of different groups according to Hb level

Variables	Overall survival			LMFS	
	Patient	5-year OS(%)	*P*	5-year LMFS	*P*
Pretreatment Hb					
Anaemia	39	61.5	0.40	58.4	0.59
No anaemia	94	58.3		53.7	
Neoadjuvant Hb					
Anaemia	99	57.9	0.11	51.8	0.14
No anaemia	34	70.2		64.6	
Adjuvant anaemia					
Anaemia	98	62.2	0.73	56.6	0.66
No anaemia	35	62.5		51.3	
Hb dynamic change					
Continuous decrease	58	52.3	0.04	49.6	0.23
Noncontinuous decrease	75	68.5		59.4	
Δ Hb level					
Δ Hb ≤ 7.6	33	75.8	0.04	62.9	0.31
Δ Hb > 7.6	100	57.5		52.6	
Pretreatment anaemia					
Δ Hb ≤ 7.6	21	77.1	0.03	62.6	0.52
Δ Hb > 7.6	18	46.5		52.9	
Pretreatment non-anaemia					
Δ Hb ≤ 7.6	12	57.7	0.91	63.6	0.53
Δ Hb > 7.6	82	53.1		52.5	
Neoadjuvant anaemia					
Δ Hb ≤ 7.6	24	76.3	0.01	64.6	0.15
Δ Hb > 7.6	75	44.7		47.4	
Neoadjuvant non-anaemia					
Δ Hb ≤ 7.6	8	57.1	0.43	57.1	0.65
Δ Hb > 7.6	26	67.4		66.5	
Adjuvant anaemia					
Δ Hb ≤ 7.6	17	19.5	0.02	69.3	0.24
Δ Hb > 7.6	81	50.1		53.4	
Adjuvant non-anaemia					
Δ Hb ≤ 7.6	15	58.6	0.85	53.8	0.80
Δ Hb > 7.6	20	58.3		50.0	

*OS* overall survival, *LMFS* lung metastasis free survival, *ΔHb* individual decreased Hb level

However, in subgroup analyses of anaemic patients with ΔHb > 7.6 in the pretreatment, neoadjuvant and adjuvant groups their 5-year OS was significantly worse than that of theiranaemic counterparts with ΔHb ≤ 7.6 (*P* = 0.03, 0.01 and 0.02, respectively).

No significant difference were found in 5-year LMFS when patients were stratified by their pretreatment, neoadjuvant, adjuvant Hb or ΔHb cut-off levels, or when patients were stratified by Hb continuous and non-continuous changes or in subgroup analysis.

### Univariate and multivariate analysis

Table 3 summarizes the univariate and multivariate analysis of common prognostic factors and Hb levels. Tumour size, alkaline phosphatase, Platelet and ΔHb status retained their prognostic value for 5-year OS in univariate and multivariate analyses.

**Table 3:** univariate and multivariate analysis of clinical factors for 5-year overall survival

Outcomes and variables	Univariate *p* value	Multivariate		
		Odds Ratios	95% CI	*P* value
5-year overall survival				
Tumor size ≦8 cm vs. >8 cm	<0.001	2.63	1.47–4.71	0.01
Age	0.337	–	–	–
Gender	0.510	–	–	–
Δ Hb ≤ 7.6 vs. > 7.6	0.039	1.99	1.20–4.46	0.044
ALP	0.035	2.13	1.35–3.37	0.040
PLT	0.042	1.99	1.01–2.19	0.050
Primary site	0.871	–	–	–
Pathological fracture	0.914	–	–	–
Histological type	0.352	–	–	–
Surgery type	0.422	–	–	–

Δ Hb: individual decreased Hb level; ALP: alkaline phosphatase; PLT: platelet

## Discussion

In this study, we investigated relationships between Hb levels during different therapy periods and survival outcomes for extremity osteosarcoma. Hb levels at diagnosis or during neoadjuvant or adjuvant chemotherapy had no predictive ability for 5-year OS or LMFS in the uni- or multivariable analyses. However, continuous Hb decrease or ΔHb > 7.6 g/L were predictive of 5-year OS in these patients. In subgroup analyses, anaemia patients whose Hb decreased > 7.6 g/L had worse survival outcomes than those whose Hb decreased < 7.6 g/L.

Anaemia has been found to reduce survival in many cancers, including cancers of cervix, head, neck, lung and breast. Published articles have indicated several underlying mechanisms. Anaemia is widely thought to lead to tumour hypoxia, and subsequently to angiogenesis, and genetic mutations, and to resistance to apoptosis, chemotherapy and radiotherapy. Green et al. reported that hypoxia would directly reduce formation of reactive O_2_ species and indirectly slow the cell cycle to affect the chemotherapeutic response [13]. Hypoxia may also decrease tumour control through the induction of hypoxia-inducible factor 1 alpha (HIF-1α), which could combine with constitutive HIF-1βto form a transcription factor that improves expression of proangiogenic agents, such as erythropoietin, vascular endothelial growth factor and glucose transporters [14]. Hypoxia is proven to increase metastasis and progression of osteosarcoma through the HIF-1α/CXCR4 pathway in vitro [15, 16]. HIF-1α also functtions in anaemia induced- doxorubicin resistance of human osteosarcoma cells [17].

Some authors suggest that anaemia is a marker for malnutrition, other comorbidities or the severity of the underlying illness [18]. Anaemia may result from bleeding, nutritional deficiencies, bone marrow damage and the malignant process itself. We may reasonably spectulate that anaemia interacts with tumour progression to affect survival outcomes of patients with malignancies.

We found no prognostic value for Hb concentrations, per se, at different treatment times on 5-year OS of osteosarcoma. However, patients with continuous Hb decreases during therapy had poorer outcomes than those without continuous Hb decreases. Furthermore, the cut-off of 7.6 g/L for decreases in Hb level (compared with initial Hb level) was a predictor of 5-year OS that we found for this population. The specificity of cut-off value for ΔHb was 31.6%, which may be caused by small amount of included patients and presence of other confounding variables, such as albumin level.

As changes in Hb levels during treatment are reportedly more important prognostic variable than the Hb level at baseline [9, 19, 20]. We surmised that a certain amount of Hb decrease would represent poorer nutrition and decreased response to CHT. However, the underlying mechanism is still unclear. Additionally, our subgroup analyses revealed that patients with pretreatment anaemia, neoadjuvant anaemia or adjuvant anaemia had poorer survival outcomes when Hb concentration decreased more than 7.6 g/L from their initial Hb concentrations. Our study did not find significant prognostic value of Hb levels on 5-year LMFS. This may reflect the insufficient number of included patients or reporting bias. The definitive effect of Hb levels on lung metastasis should be studied in future clinical trials.

Correction of anaemia should be considered in clinical practice as decreased Hb levels by more than 7.6 g/L apparently affect treatment outcomes in extremity osteosarcoma. To our knowledge, blood transfusion and erythropoietin could be used to correct anaemia. Kapp et al. found that transfusion of red blood cells could quickly improve Hb concentrations and prognosis in anaemia patients who underwent radiotherapy for cervical cancer [21]. The latest prospective, randomized controlled trial revealed that patients with cancers who received a restrictive strategy of erythrocyte transfusion (transfusion when haemoglobin concentration < 7 g/dl) had more mortality and severe clinical complications than those receiving a more liberal strategy (transfusion when haemoglobin concentration < 9 g/dl) [22]. This study indicates the increased necessity of anaemia rectification. However, some studies reported a negative effect of blood transfusion in anaemia patients who underwent radiotherapy for cervical carcinoma, possibly because of immunosuppression and other adverse effects [23, 24]. In recent years, erythropoietin has been extensively used in the clinic. Some studies have shown that in patients with oesophageal cancers, adminstration of erythropoietin led to lower transfusion requirements and a greater survival benefit than placebo [25]. Neverthelss, a prospective, randomized-controlled trial of 351 patients with head and neck cancers found that patients who received erythropoietin to achieve Hb levels higher than 140 g/L (women) or 150 g/L (men) had poorer locoregional progression-free survival than those who received placebo [26]. The roles of blood transfusion and erythropoietin thus remain controversial. Few data are available on the effect of anaemia correction for osteosarcoma patients. Therefore, future studies on how to correct anaemia for patients who suffer from cancers, including osteosarocma, are needed.

The strengths of our study include a cohort who received standard treatments and long follow-up (more than 5 years); a focus on the prognostic value of dynamic changes in Hb levels during treatments, and the use of multivariate methods of statistical analysis to test the prognostic value of Hb level change during therapy. Our study also had some limitations. Firstly, it was a retrospective study, including data from only one institution. Secondly, the number of patients included was not large enough to draw concrete conclusions regarding differences between treatment groups. Thirdly, continuous haemoglobin decrease can only be evaluated at the conclusion of treatment according to the data analysis regimen of this study.

## Conclusion

In our study, Hb levels at the time of diagnosis, neoadjuvant or adjuvant chemotherapy cannot predict 5-year OS and LMFS on Enneking stage IIB extremity osteosarcoma. However, patients with continuous Hb decrease were shown to have worse 5-year OS than those without continuous Hb decrease. Patients whose Hb concentration decreased by more than 7.6 g/L had worse outcomes than patients whose Hb decreased by less. The role of anarmia and the effects of attempts to correct it, in outcomes for osteosarcoma patients should be investigated in future trials.

## Abbreviation

Hb: haemoglobin
LMFS: lung metastasis free survival
OS: overall survival
ROC: operating characteristic
Δ Hb: difference in Hb decrease
